# Characterization and quantification of iron species in the banded iron formations (BIFs) in China Craton to explore the potential for H_2_ production using XRD and Mössbauer spectroscopy

**DOI:** 10.1371/journal.pone.0316540

**Published:** 2025-01-24

**Authors:** Hyo-Im Kim, Inkyeong Moon, Minkyeong Kim, Hyuk Jun Lee, Hyunkyung Choi, Young Rang Uhm, Lei Liu, Jonguk Kim, Wonnyon Kim

**Affiliations:** 1 Department of Geology, Gyeongsang National University, Jinju, Republic of Korea; 2 Research Institute of Molecular Alchemy, Gyeongsang National University, Jinju, Republic of Korea; 3 Ocean Georesources Research Department, Korea Institute of Ocean Science and Technology, Busan, Republic of Korea; 4 HANARO Utilization Division, Korea Atomic Energy Research Institute, Daejeon, Republic of Korea; 5 School of Geosciences and Info-Physics, Central South University, Changsha, China; Ural Federal University named after the first President of Russia B N Yeltsin Institute of Physics and Technology: Ural’skij federal’nyj universitet imeni pervogo Prezidenta Rossii B N El’cina Fiziko-tehnologiceskij institut, RUSSIAN FEDERATION

## Abstract

Banded iron formations (BIFs), significant iron ore deposits formed approximately 2.3 billion years ago under low-oxygen conditions, have recently gained attention as potential geological sources for evaluating hydrogen (H₂) production. BIFs are characterized by high concentrations of iron oxide (20 to 40 wt.%) and low Fe^3^⁺/Fe_tot_ ratios, representing a major source of ferrous iron on Earth. This study investigates the mineralogical and geochemical characteristics of iron ore samples from the Wugang and Hengyang BIFs in China using X-ray diffraction (XRD) and Mössbauer spectroscopy to examine H_2_ generation potential. XRD analysis and microscopic observations showed that the magnetite and hematite are the primary ore minerals in BIFs in China Craton. Mössbauer spectroscopic results provided the quantified information on the fractions of each iron species in varying minerals. Particularly, the Fe^3+^ tetrahedral sites and octahedral sites occupied by both Fe^2+^ and Fe^3+^ in magnetite and Fe^3+^ octahedral sites in hematite were determined. We estimated H₂ production potential by calculating the relative fraction of Fe^2+^ in magnetite relative to total number of iron atoms in the bulk samples from the Mössbauer results. The pyroxene-bearing BIF in Wugang (P-BIF) contains magnetite predominantly (~30.4 wt%), and the fraction of Fe^2+^ in magnetite is ~26%. Based on the quantified values, the maximum potential for H_2_ generation from P-BIF in Wugang could be ~630 mmol H₂/kg rock. Due to the variation of mineralogical composition depending on the types and locations of occurrence of BIF, the H_2_ generation potential also varies. For example, contrast to P-BIF in Wugang, the hematite-rich BIF from Hengyang, containing ~6.0 wt% of magnetite, showed significantly lower Fe^2+^ fraction in magnetite (~5%), resulting in low H_2_ potential (~120 mmol H₂/kg rock). This study presents that a prevalence of magnetite in BIFs has considerable potential for H₂ production due to low Fe^3+^/Fe_tot_, suggesting that the magnetite-rich iron ore can be effectively utilized as the source of stimulated hydrogen production. The current results also highlight that the Mössbauer spectroscopy is essential to provide the database of relative fractions for each iron species in BIFs, which allows us to estimate the quantity of H_2_ released from BIFs.

## Introduction

Explorations of geological hydrogen (H_2_) in diverse settings have garnered tremendous attention due to its potential to provide a sustainable and clean energy source as a promising alternative to fossil fuels [1–3]. Recent studies have discovered large accumulations and releases of *natural* H_2_ in regions such as Mali in Africa [4], Oman in West Asia [5–7], Kansas in United States [8], and etc. [3]. Along with the exploration of natural H_2_, the efforts to proactively generate H_2_ from geological source rock (i.e., *stimulated geological hydrogen*) have been initiated to facilitate the large-scale and sustainable hydrogen production [6, 9].

The oxidation of Fe^2+^ in the iron-rich rocks via water-rock interaction is considered a key process to in both natural H_2_ and generating H_2_ [10, 11]. Particularly, serpentinization, a well-known geological process that produces natural H_2_ in association with the mafic and ultramafic rocks, occurs when Fe^2+^ in ferromagnesian minerals (such as olivine, pyroxene, and etc.) is oxidized to Fe^3+^, resulting in the reduction of H_2_O [7, 12, 13]. Due to the significance of serpentinization, the previous and ongoing explorations for naturally released H_2_ have focused on the geological contexts related to tectonic boundaries associated with oceanic lithosphere and mantle rocks such as ophiolite system [7, 14–16], mid-ocean ridges [17–19], and active vents [20–22]. In this context, the injection of fluid into rock containing Fe^2+^-bearing minerals can promote the oxidation reactions that involve H_2_ emission, with chemical reaction of 2FeO_(rock)_ + H_2_O → Fe_2_O_3(rock)_ + H_2_ [9, 23]. By constraining the geochemical conditions of fluids, the production of H_2_ from the source rocks can become more efficient. For the determination of the source rock for obtaining H_2_, the accurate description of geological targeted rocks including mineralogical identification and quantification of iron-bearing oxide minerals are necessary.

Recently, banded iron formations (BIFs) have re-emerged as a source rock for production of H_2_ [24, 25] due to the prevalence of iron oxide phases. BIFs, which consist primarily of iron oxide minerals such as magnetite and hematite with high total Fe_2_O_3_ contents (~20–50 wt%), formed during the Precambrian era between 3.8 and 1.8 billion years ago [11] through the oxidation of dissolved Fe^2+^ (aq) in the oceanic water into Fe^3+^ [26]. The mineral phases constituting BIFs vary depending on the redox conditions, chemical compositions, material sources and microbial activities of the ancient aqueous system. Meanwhile, magnetite (Fe_3_O_4_) is considered to be a key mineral involved in the generation of H_2_: magnetite has an inverse-spinel structure characterized by the general stoichiometry (Fe^3+^)^A^[Fe^2+^Fe^3+^]^B^O_4_, where A sites are tetrahedrally coordinated (Td^M^) and B sites are octahedrally coordinated (Oh^M^), which are randomly occupied by approximately equal numbers of Fe^3+^ and Fe^2+^ [27, 28].

Until recently, magnetite was regarded as a by-product of the serpentinization process, resulting from the alteration of Fe^2+^-bearing silicate minerals to Fe^3+^-bearing serpentine and/or catalysts of H_2_-production reactions [11, 12, 24, 29, 30]. A recent study suggests that the presence of magnetite in reactants (i.e., source rock) can promote oxidation reactions under low-temperature conditions due to its spinel crystal structure, which facilitates electron transfer on the octahedron sites in spinel structures [30]. Taking into account the stability of BIFs which formed in the Precambrian era, the formation of large-scale accumulation of naturally generated H_2_ from BIFs is considered to be kinetically unfavored in the ambient condition. However, the experimental studies for water-rock interaction using magnetite on low-temperature conditions (under 200°C) controlling the temperature, pH, specific surface area and mineral composition of source rock showed that considerable amount of H_2_ can be released from magnetite [24, 29, 30]: for example, about 6.3 μmol/g of H_2_ were generated from natural magnetite at the temperature of ~5–20°C [29]. These previous results suggest that the magnetite could be a promising candidate for H_2_ production with low amount of energy consumption during the engineering process for stimulated geological hydrogen. Therefore, the BIFs, which are abundant in magnetite and contain high total Fe content, could be deliberated its potential as a source for H_2_ generation.

The application of the natural magnetite for hydrogen production offers several advantages compared to other methods. Unlike conventional approaches such as water electrolysis and fossil fuel reforming, the development of stimulated hydrogen production using magnetite can significantly reduce energy consumption and CO_2_ emission during H_2_ production process, contributing to a carbon-neutral and environmentally sustainable process. Moreover, magnetite-based H_2_ production methods are potentially more reliable and consistent than H_2_ production from biomass and solar photocatalysis, given the widespread availability of magnetite in various geological settings [24, 25]. While it is necessary to study the efficient conditions for H_2_ generation from magnetite and identify suitable geological sites to achieve cost-effectiveness, H_2_ production using magnetite in natural rock samples remains a highly promising approach, considering these advantages.

To evaluate the potential of BIFs as a source rock of H_2_, the possible amount of H_2_ release should be estimated based on the geochemical and mineralogical characterizations. Specifically, identification of iron-bearing mineral phases and quantification of their fractions in BIFs are necessary to determine the Fe^2+^ contents which can be involved in the oxidation reaction that produces H_2_. X-ray diffraction (XRD) is an effective experimental tool for identifying the mineral species in the bulk rock samples, thus, previous studies have utilized XRD to investigate the mineral compositions in BIFs from diverse regions [31–33]. To better constrain the varying iron species, Mössbauer spectroscopy which a powerful technique for quantifying crystallographically distinct iron sites in diverse phases is necessary [34, 35]. This nuclear spectroscopic technique has widely utilized in the studies of natural and synthetic materials such as volcanic rocks in Earth and Mars [36–39], silicate glasses [40, 41], and BIFs [33, 42, 43] via quantifying the relative fractions of iron-bearing phases including magnetite and hematite.

Mössbauer spectroscopy which has been applied to diverse BIFs from various depositional terrains (e.g., West Greenland, Canada, Australia, North China and etc.) provides detailed insights into the paleoenvironmental conditions during BIF formation, diagenetic and/or metamorphic pathways, and the degree of weathering [33, 44, 45]. More recently, a study utilizing Mössbauer spectroscopy attempted to quantify the mineral species in BIF samples from the Pilbara Craton in Australia to calculate the potential of H_2_ production [25]. To identify suitable BIFs for H_2_ production, the database of the quantified magnetite contents in diverse natural BIFs are required.

In this paper, we performed XRD and Mössbauer spectroscopy to identify and quantify the iron-bearing mineral phases in BIFs from the Wugang and Hengyang, located in the North and South China Cratons, respectively. It is well known that the North China Craton (NCC) contains primarily Neoarchean-Paleoproterozoic BIF deposits and the BIF types vary with the specific locations [46–48]. Although the details of BIFs in South China Craton (SCC) remain to be explored, a recent study has reported that Mesoarchean BIFs are deposited in the SCC [49]. This study provides the database of relative fractions for each iron species in BIFs, which allows us to estimate the quantity of H_2_ released from BIFs. We then discuss the potential of BIFs in China Craton as the source of H_2_ production.

### Geological setting

**Fig 1:**
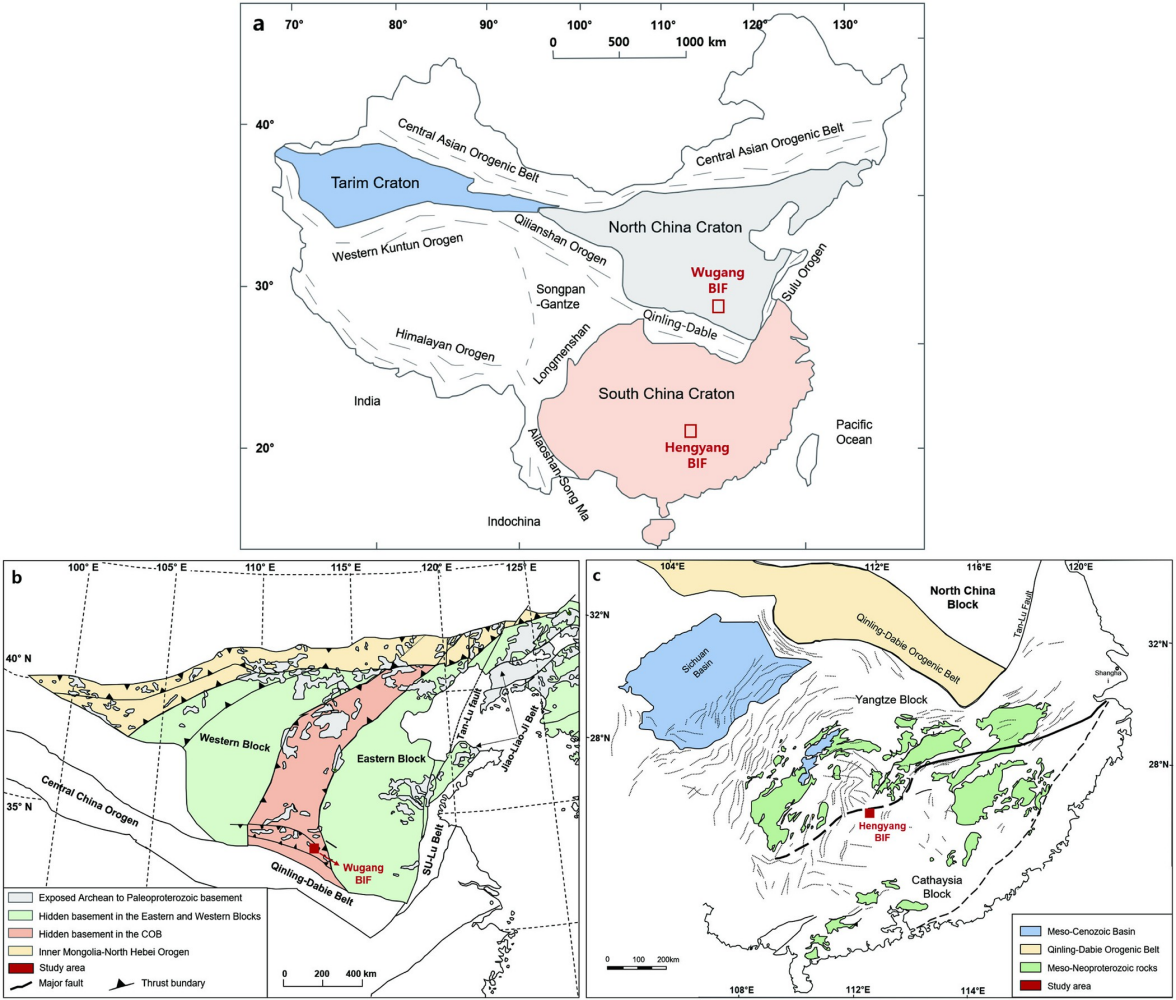
Geological map of the study area. (a) The schematic tectonic map of China showing the major Precambrian blocks [North China Craton (NCC) and South China Craton (SCC)] [63]. Geological maps of (b) NCC [64, 65] and (c) SCC [66] with sampling locations of the Wugang BIF within the NCC and the Hengyang BIF within the SCC.

China hosts three major Precambrian cratons: The NCC, the SCC, and the Tarim Craton (Fig 1A), each with a geological history spanning billions of years [50]. NCC is consist of Archean to Paleoproterozoic metamorphosed basement with Mesoproterozoic to Phanerozoic unmetamorphosed sedimentary cover. The basement rock of craton is primarily composed of Neoarchean tonalitic–trondhjemitic–granodioritic (TTG) gneisses and metamorphosed supracrustal rocks. In contrast, Paleoarchean and Mesoarchean rocks are largely confined to the eastern regions of the craton, particularly in eastern Hebei Province and Anshan in Liaoning Province, where rocks dating back to 3.85 billion years have been identified [51–53] (Fig 1B). The South China Craton is partitioned into two distinct geological blocks: the Yangtze Block in the north and the Cathaysia Block in the south, each characterized by unique crustal ages and tectonic histories [42, 54–56]. The basement of the Yangtze Block is composed of Archean rocks [57, 58], whereas the Cathaysian Block primarily contains Paleo-Mesoproterozoic rocks with some Late Archean elements [59]. The collision between these two blocks led to the Sibao Orogeny, dated between 0.9 and 1.3 billion years ago, which aligns with the global Grenville orogeny [60–62] (Fig 1C).

In this study, the iron ore samples were collected from the Wugang BIF in the North China Craton (NCC) and the Hengyang BIF in the South China Craton (SCC) (Fig 1). The Wugang BIF is located in the Neoarchean Taihua complex in the Wugang area and is considered one of the representative iron ores in the Central Plains [48]. The Taihua complex is discretely exposed along the southern margin of the north-south striking TNCO and consists of early Precambrian medium-high grade metamorphic rocks with TTG gneisses, amphibolites, supracrustal rocks and so forth. The area is primarily comprising the Neoarchean blocks of Huashan, Xiaoshan, Luoning, Lushan, and Wugang from northwest to southeast. Among the blocks, BIFs are distributed in the Wugang and Lushan areas. In this study, we focused Wugang BIF which possessed more than 600 million tons. The iron source was provided by mixture of high-T hydrothermal fluid and seawater with negligible effect of detrital contamination in an anoxic marine environment [64, 67]. Detrital zircon U-Pb dating provides an age constraint between 2.40 and 2.47 Ga [54]. Combined the features of geological condition, related host rock, and geochemical data, Wugang BIF have Superior-type affinity and deposited in near-shore continental-shelf or back-arc basin environments [48].

The Hengyang basins are located within the South China Craton, at the border between the Yangtze Block and the Cathaysian Block [68]. The geological data for the Hengyang BIF has only been documented in domestic publications. This study area is located in the central segment of the Qin-Hang Belt, adjacent to the western part of the Hengyang Basin. The region features a complex geological structure, with exposed strata including the Proterozoic Nanhuan Group, Sinian System, Cambrian and Ordovician systems from the Lower Paleozoic, and the Devonian, Carboniferous, and Permian systems from the Upper Paleozoic, as well as the Triassic, Jurassic, Cretaceous systems from the Mesozoic, and Quaternary deposits from the Cenozoic [69]. The samples are from the Fulu Formation of the Gaojian Group within the Nanhua System, characterized by a marine transgression from shallow to deep water (720–635 Ma). Stratigraphic evidence from drilling supports the assignment to the Fulu Formation, showing consistent thickness despite faulting (unpublished data).

## Methods

### Samples

A total of 10 samples, including four iron ore samples from the Wugang BIF, two paragneiss samples associated with Wugang BIF, and four iron ore samples from the Hengyang BIF, were selected for this study. The samples were collected from natural outcrops of the BIFs, which were exposed at the surface, enabling easy access for direct sampling without the need for excavation or drilling. Since the BIFs are not operational, the iron ore sampling was conducted without a permit. The analysis for chemical composition of bulk rocks and XRD experiments were conducted on all 10 samples. For the Mössbauer experiments, three samples from Wugang and two samples from Hengyang with high Fe_2_O_3_ contents (> 30 wt%), representative of each petrological characteristic, were chosen.

### Whole-rock analysis

Whole rock analysis was performed by at Activation Laboratories (Actlabs Canada) with “4 Litho” research analytical protocol. Major and trace elements were analyzed by Fusion ICP-OES (with an error range of ± ~0.1 wt%) and Fusion ICP-MS (with an error range of ± ~2%), respectively (Table 1). Note that the total values of major elements obtained by ICP-OES may exceed or fall short of 100% due to intrinsic factors during experiment such as presence of volatiles, variations of metal oxidation states, and matrix effects. Total values of major elements within the range of 99–101% are considered acceptable.

**Table 1:** Whole-rock compositions of the BIFs and paragneiss.

Region	Wugang (WG)						Hengyang (MC)				
Sample	20WG-24-a	20WG-24-b	20WG-23-2-a	20WG-23-2-b	20WG-21-a	20WG-21-b	23MC-3-a		23MC-3-b	23MC-2-A-a	23MC-2-A-b
Rock-type	P-BIF	P-BIF	Q-BIF	Q-BIF	Paragneiss	Paragneiss	BIF		BIF	BIF	BIF
*Major element (wt%)*											
SiO_2_	53.4	51.29	41.96	43.79	69.67	68.25	62.12		61.99	49.7	72.08
Al_2_O_3_	0.13	0.13	0.95	1.1	14.59	14.4	4.21		4.46	4.2	6.57
Fe_2_O_3_^(T)^	33	38.59	49.1	44.55	3.82	4.36	30.63		30.43	40.42	11.71
MnO	0.227	0.205	0.088	0.094	0.102	0.111	0.061		0.064	0.126	0.2
MgO	3.66	3.16	5.43	4.93	2.8	2.94	0.99		1.11	0.98	1.49
CaO	9.4	8.08	1.18	1.63	2.19	2.22	0.55		0.62	1.84	3.45
Na_2_O	0.62	0.51	0.03	0.08	2.26	2.05		0.13	0.12	0.04	0.14
K_2_O	< 0.01	< 0.01	< 0.01	0.04	2.81	2.72	1.23		1.34	1.45	2.18
TiO_2_	0.004	0.005	0.033	0.03	0.611	0.629	0.365		0.405	0.245	0.35
P_2_O_5_	0.04	0.05	0.03	0.07	0.12	0.11	0.13		0.17	0.14	0.35
LOI	-1.1	-1.21	1.6	1.97	1.83	2.07	-0.19		-0.1	0.73	1.32
Total	99.4	100.8	100.4	98.27	100.8	99.87	100.2		100.6	100.8	99.83
*Trace elements (ppm)*											
Sc	< 1	< 1	< 1	< 1	13	16	8		8	6	10
Be	< 1	< 1	< 1	< 1	< 1	< 1	< 1		1	< 1	1
V	< 5	< 5	7	16	73	74	62		62	77	41
Ba	< 2	< 2	5	6	276	267	578		616	648	998
Sr	16	14	8	12	164	159	43		51	193	260
Y	2	2	2	2	13	16	17		29	14	25
Zr	5	4	8	8	194	191	153		189	50	144
Cr	< 20	< 20	< 20	< 20	< 20	40	70		80	30	80
Co	< 1	< 1	2	2	6	7	2		2	4	3
Ni	< 20	< 20	< 20	< 20	< 20	20	< 20		< 20	< 20	< 20
Cu	< 10	< 10	< 10	< 10	< 10	10	< 10		< 10	< 10	< 10
Zn	< 30	< 30	< 30	< 30	90	90	< 30		< 30	40	40
Ga	< 1	< 1	2	2	18	18	6		6	7	8
Ge	4	4	4	3	< 1	< 1	2		2	2	4
As	< 5	< 5	< 5	< 5	< 5	< 5	< 5		< 5	< 5	< 5
Rb	< 2	< 2	< 2	< 2	112	108	34		39	38	57
Nb	< 1	< 1	1	2	6	6	8		10	6	8
Mo	< 2	< 2	< 2	< 2	< 2	< 2	< 2		< 2	< 2	< 2
Ag	< 0.5	< 0.5	< 0.5	< 0.5	< 0.5	< 0.5	< 0.5		< 0.5	< 0.5	< 0.5
In	< 0.2	< 0.2	< 0.2	< 0.2	< 0.2	< 0.2	< 0.2		< 0.2	< 0.2	< 0.2
Sn	< 1	1	1	1	3	3	1		1	2	1
Sb	< 0.5	< 0.5	< 0.5	< 0.5	< 0.5	< 0.5	3.4		3	8.1	7.9
Cs	< 0.5	< 0.5	1	0.8	3	3.2	2.5		2.8	0.5	0.8
La	1.4	1.3	2.1	2.8	24.5	26.4	16.4		17.9	9.9	19.8
Ce	1.8	1.6	3.7	4.9	49.6	53.2	31.1		35.1	22.2	41.2
Pr	0.17	0.14	0.4	0.52	5.96	6.32	3.85		4.09	2.83	5.06
Nd	0.6	0.5	1.4	1.8	22.5	24	15.1		16.6	12.1	21.4
Sm	0.2	< 0.1	0.3	0.4	4.1	4	3.2		3.7	2.6	4.6
Eu	0.05	0.05	0.08	0.12	0.94	0.92	0.58		0.73	0.44	0.81
Gd	0.1	0.1	0.2	0.3	3	3.2	2.7		3.6	2.4	4.1
Tb	< 0.1	< 0.1	< 0.1	< 0.1	0.4	0.5	0.4		0.7	0.4	0.6
Dy	0.2	0.1	0.3	0.3	2.7	3	2.9		4.7	2.7	4.5
Ho	< 0.1	< 0.1	< 0.1	< 0.1	0.5	0.6	0.6		1.1	0.6	1
Er	0.1	0.1	0.2	0.2	1.4	1.9	2.3		3.5	1.6	3
Tm	< 0.05	< 0.05	< 0.05	< 0.05	0.19	0.3	0.38		0.55	0.25	0.46
Yb	0.1	0.1	0.2	0.2	1.4	2.1	2.8		4	1.7	3.3
Lu	0.02	0.02	0.03	0.03	0.23	0.33	0.47		0.64	0.26	0.57
Hf	< 0.2	< 0.2	< 0.2	< 0.2	4.6	4.7	4		4.7	1.4	3.3
Ta	< 0.1	< 0.1	< 0.1	< 0.1	0.3	0.3	0.3		0.4	0.3	0.5
W	< 1	< 1	< 1	2	1	< 1	3		< 1	1	< 1
Tl	< 0.1	< 0.1	< 0.1	< 0.1	0.4	0.4	0.1		0.1	0.1	0.2
Pb	< 5	< 5	< 5	< 5	6	7	12		13	8	8
Bi	< 0.4	< 0.4	< 0.4	< 0.4	< 0.4	< 0.4	< 0.4		< 0.4	< 0.4	< 0.4
Th	0.2	0.2	0.3	0.4	4.2	3.9	4.3		4.6	3.6	5.4
U	0.2	0.1	0.3	0.4	0.4	0.4	0.4		0.5	0.5	0.4

Note: The error range for major elements analyzed by ICP-OES is approximately ±0.1 wt%, while for trace elements analyzed by ICP-MS, it is approximately ±2%.

### X-ray diffraction

X-ray diffraction (XRD) patterns for BIF samples including two paragneiss samples associated with Wugang BIF were collected on Bruker D8 advance X-ray diffractometer at the Department of Geology, Gyeongsang National University. The Cu Kα X-ray source (λ = 1.541 Å) with a voltage of 40 kV and a current of 40 mA was used to obtain the diffraction patterns ranging from 10° to 80° with a step size of 0.02°, and a step time of 0.4 s.

### Mössbauer spectroscopy

The detailed description of iron oxide species in the BIF samples were analyzed using the room-temperature ^57^Fe resonant absorption Mössbauer spectrometer in Korea Atomic Energy Research Institute (KAERI). Five powdered samples with high Fe_2_O_3_ contents (over 30 wt%) were prepared for the Mössbauer experiment. The Mössbauer experiments were conducted with a radioactivity of 50 mCi ^57^Co/Rh source operating in the constant acceleration mode between -12 and 12 mm/s in transmission geometry. The spectrometer was calibrated with a 4 μm-thick α-Fe foil as the reference absorber. The isomer shifts (δ, mm/s) are reported relative to α-Fe metal at room temperature (298 K).

## Result and discussion

### Sample description

**Fig 2:**
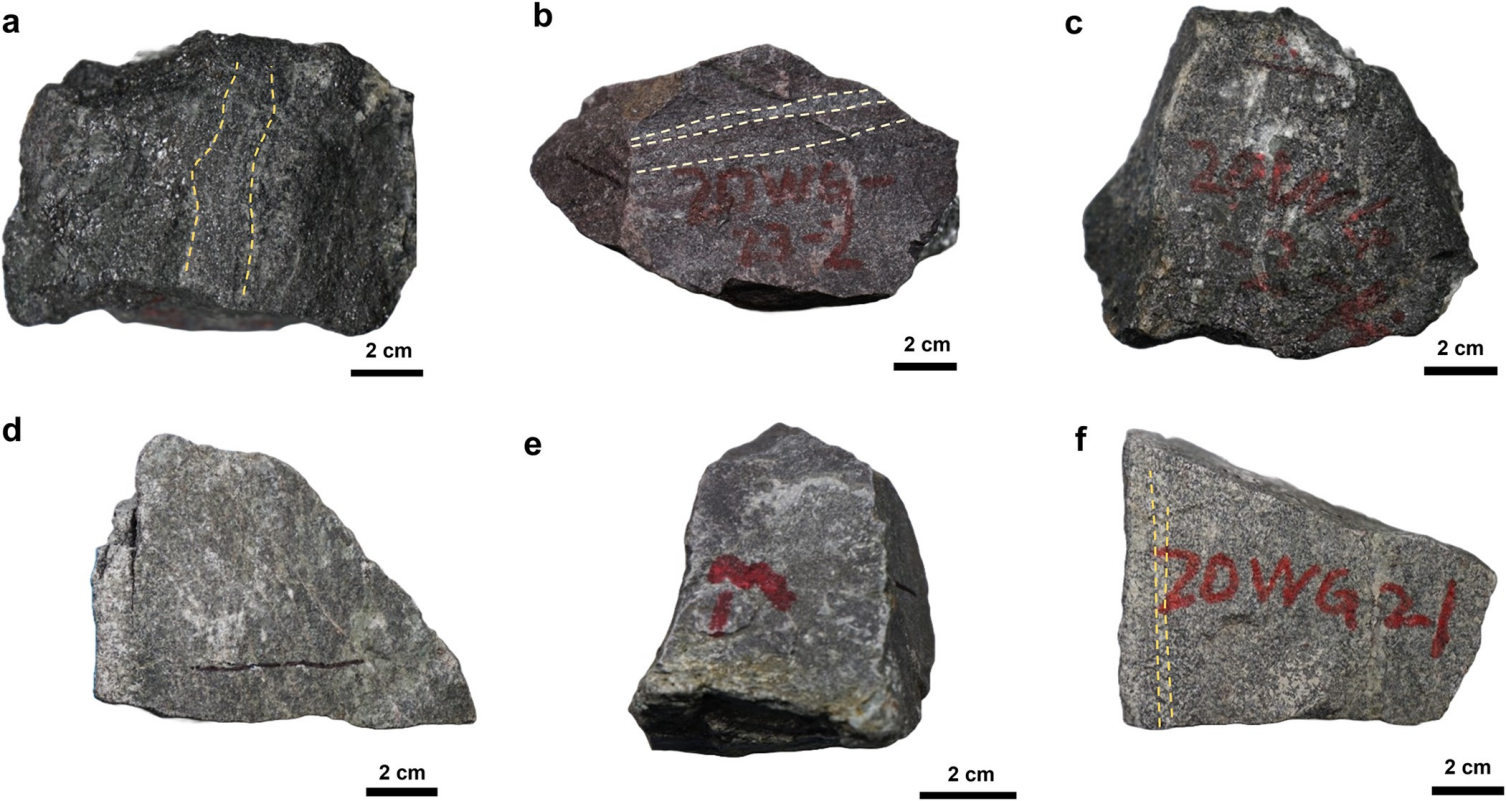
Representative photographs of samples used in this study. (a-c) Wugang BIFs. (d-e) Hengyang BIFs. (f) paragneiss associated with Wugang BIF. The iron ores from samples (a, b) exhibit a distinctive layered texture. The darker layers are composed of magnetite and pyroxene, while the lighter layers consist of quartz. In contrast, the remaining ores exhibit a massive texture (c-e).

**Fig 3:**
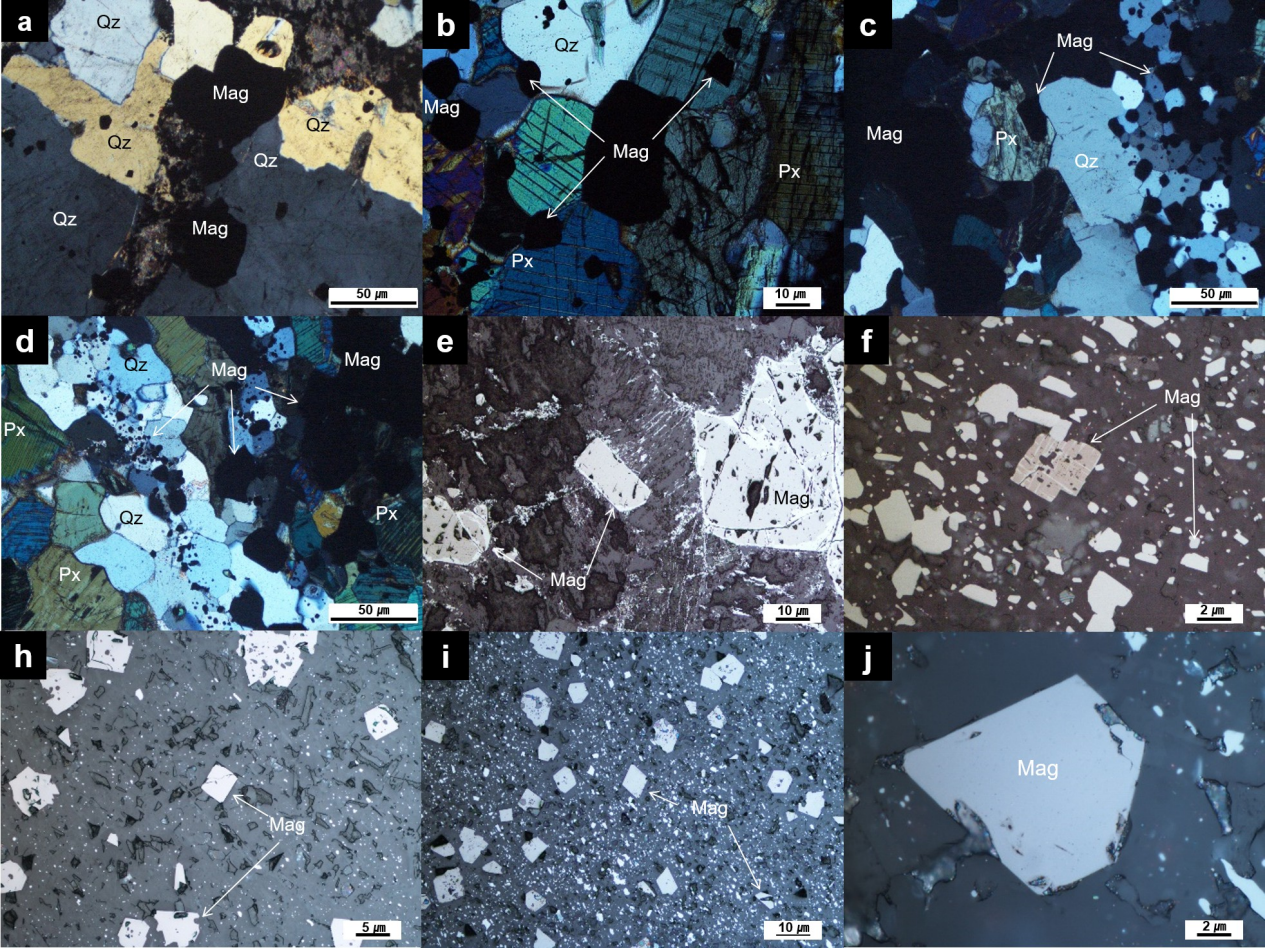
Representative photomicrographs of the iron ores from Wugang BIF (a-d) and Hengyang BIF (e-j). (a-d) Coarse-grained magnetite and quartz with minor pyroxene. (e-f) Photomicrograph of relict of magnetite replaced by hematite and (h-i) subhedral to euhedral magnetite with sizes of up to 40 μm. Abbreviations: Mag: magnetite; Px: pyroxene; Qz: quartz.

The iron ores from the Wugang BIF have relict layering texture (Fig 2A and 2B) and massive texture (Fig 2C) are primarily composed of magnetite, hematite, and quartz, with minor amounts of clinopyroxene and plagioclase, and rare occurrences of talc. Under microscopic observation, quartz occurs as irregular aggregates, displaying subhedral to anhedral forms, with sizes up to approximately 60 μm. Additionally, magnetite appears as single anhedral crystals or aggregates ranging from 10 to 60 μm in size (Fig 3A–3D). The iron ore from Hengyang BIF exhibit massive texture (Fig 2D and 2E) and are composed of magnetite, hematite, quartz with minor K-feldspar. The magnetite displays euhedral to subhedral shapes, with sizes less than 20 μm (Fig 3E–3F). In particular, the magnetite in Hengyang BIF is disseminated and partially replaced by hematite with white-pinkish oxide stripped by brighter edge, indicative of an oxidation process (Fig 3E and 3F).

### Chemical compositions: Major and trace elements

Table 1 shows the major and trace element composition of 8 BIF samples, comprising 4 samples from Wugang (WG) and 4 samples from Hengyang (MC). The chemical compositions of two paragneiss samples associated with the Wugang BIFs are also presented. The Wugang BIF displays the typical characteristics of Archean BIF, composed of high SiO_2_ + Fe_2_O_3_^T^ content of 86.4–91.1 wt%. Note that Fe_2_O_3_^T^ refers the weight percent of total iron oxide. The major compositions of Wugang BIF samples in the current study are largely consistent with previously reported values [48, 70]. The Wugang BIFs have two different types of iron ores: pyroxene-rich BIF (i.e., P-BIF) and quartz-rich BIF (i.e., Q-BIF). In the outcrop, P-BIFs are located in the outer part of the ore body, while Q-BIFs are found in the central part [49]. The Wugang P-BIF samples contain relatively higher SiO_2_ (51.3–53.4 wt%) and lower Fe_2_O_3_^T^ contents (33.0–38.6 wt%), compared to the Wugang Q-BIF samples which have SiO_2_ contents with 42.0–43.8 wt% and Fe_2_O_3_^T^ contents with 44.6–49.1 wt%. The CaO contents in P-BIFs (8.1–9.4 wt%) are significantly higher than those in Q-BIFs (1.2–1.6 wt%), indicating the presence of Ca-rich pyroxene in the P-BIF samples, as shown in the XRD results (see Section ‘Major mineral phases: XRD results’). In both Wugang BIF samples, Na_2_O, K_2_O, and TiO_2_ contents are under 1 wt%. Meanwhile, the paragneiss samples obtained from the vicinity of Wugang BIFs exhibit low Fe_2_O_3_^T^ contents of approximately 3–4 wt%, while their Al_2_O_3_ contents are significantly higher (~14.5 wt%), with respect to the BIF samples (0.13–1.1 wt%).

The Hengyang BIF samples have high Fe_2_O_3_^T^ contents of 30.4–40.4 wt% with SiO_2_ contents of 49.7–62.1 wt%, while the one sample unit shows considerably low Fe_2_O_3_^T^ content of 11.7 wt% and high SiO_2_ content of 72.1 wt%. The Al_2_O_3_ contents of Hengyang BIF samples (> 4 wt%) are notably higher than those of Wugang BIF samples (< 1 wt%), suggesting the possibility of crustal contamination [71] in Hengyang BIF.

The trace and rare earth element (REE) compositions of the BIF samples show distinct differences depending on the sampling locations. For Wugang BIF samples, the contents of trace and REE elements are mostly below the detection limit, regardless of the type. The lack of trace elements such as Hf, Zr, and Sc indicates that the involvement of clastic detritus into BIFs can be negligible in the Wugang area [72]. The remarkably high Sr contents (8–16 ppm) are consistent with the previously reported values [48]. On the other hand, the contents of trace and REE elements in the Hengyang BIF samples are significantly higher than those in Wugang BIFs. The total REE concentrations in the Hengyang BIF range from approximately 83 to 110 ppm, whereas those in the Wugang BIF ranges from about 4 to 12 ppm, indicating a distinct difference in the formation mechanisms between the Hengyang and Wugang BIFs. In particular, the remarkable high contents of Hf (1.4–4.7 ppm), Zr (50–189 ppm), and Sr (6–10 ppm) suggest significant contamination from terrigenous inputs into Hengyang BIFs.

### Major mineral phases: XRD results

**Fig 4:**
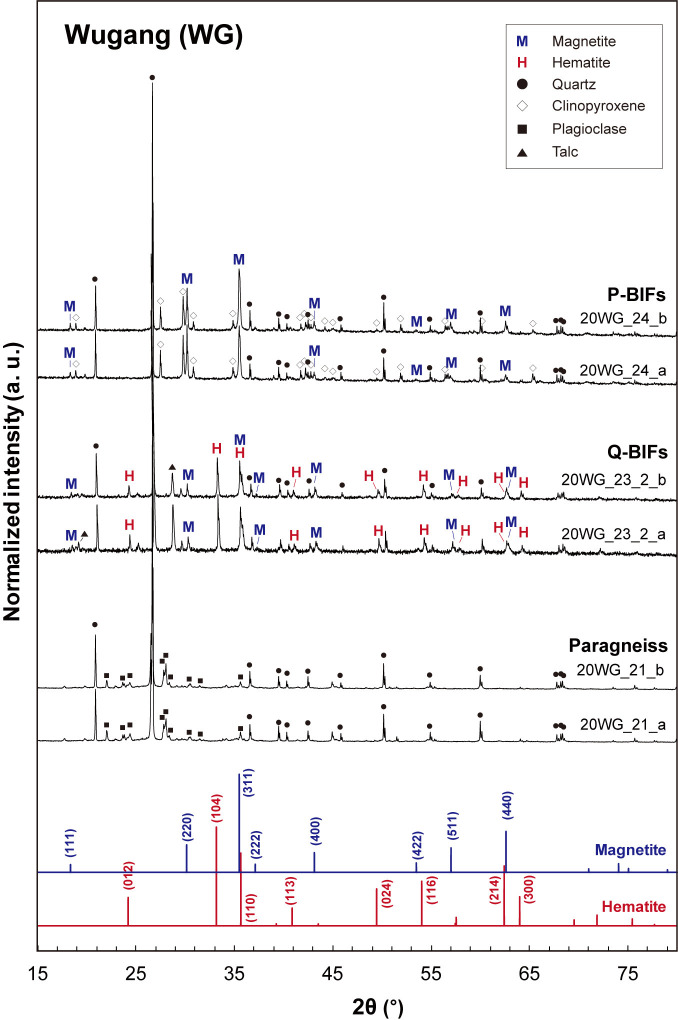
XRD patterns for BIF and paragneiss samples from Wugang (WG) region. M and H refers to magnetite (JCPDS No. 19–0629) and hematite (JCPDS No. 33–0664), respectively (closed circles: quartz [76]; open diamonds: clinopyroxene [75]; closed squares: plagioclase [80]; closed triangles: talc [81]).

**Fig 5:**
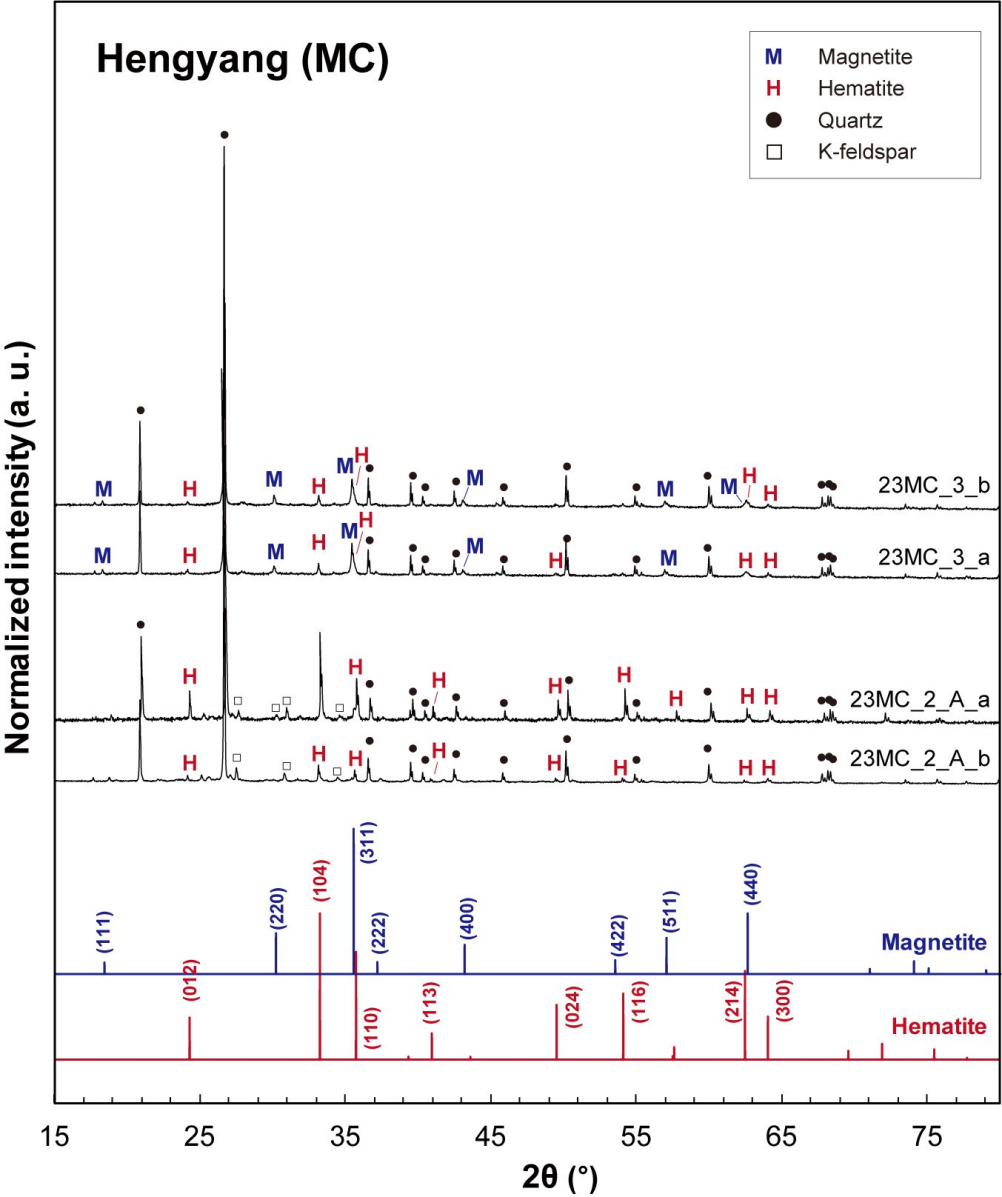
XRD patterns for BIF and paragneiss samples from Hengyang (MC) regions. M and H refers to magnetite (JCPDS No. 19–0629) and hematite (JCPDS No. 33–0664), respectively (closed circles: quartz [76] and open squares: K-feldspar [79]).

Fig 4 presents the X-ray diffraction (XRD) patterns for Wugang BIF and two associated paragneiss samples, showing the predominance of quartz in all samples and the presence of iron oxide minerals [i.e., magnetite (Fe_3_O_4_) and hematite (Fe_2_O_3_)] in BIF samples, consistent with the microscopic observations. The XRD patterns for P-BIFs exhibit the distinct diffraction peaks corresponding to magnetite (JCPDS No. 19–0629), with the main peak occurring at a 2θ of ~36° for the (311) plane [73, 74]. Additionally, the clinopyroxene [i.e., augite, (Ca,Mg)_2_Si_2_O_6_] is observed in XRD patterns for P-BIF [75] with quartz [76]. While the diffraction peaks for magnetite are clear and evident, those for hematite is absent in the XRD patterns for P-BIF, indicating that the magnetite is major iron oxide phase in P-BIFs. For Q-BIFs, the coexistence of hematite and magnetite is confirmed in the XRD patterns. Note that the main diffraction peak of hematite appears at a 2θ of ~33° for the (104) plane (JCPDS No. 33–0664) [77, 78]. The paragneiss samples associated with the Wugang BIFs are primarily composed of quartz and plagioclase, with no other iron oxide phases observed in the XRD patterns. The XRD patterns for BIF samples from Hengyang are shown in Fig 5. Two samples collected from Hengyang basin (23MC-3-a and 23MC-3-b) contain both magnetite and hematite as the iron oxide phase, with the quartz as a dominant mineral phase. The other two samples (23MC-2-A-a and 23MC-2-A-b) contain the quartz and hematite with K-feldspar [79] as a minor phase. In these samples, the diffraction patterns for magnetite were not observed.

By combining microscopic observation with XRD analysis, magnetite is dominant in Wugang BIF, whereas hematite is primary phases in the Hengyang BIF. Since the presence of Fe^2+^ in BIF is essential to produce H_2_ during alteration and hydration processes, the relative fraction of magnetite can be a major factor in estimating the amount of H_2_ released from BIF. Therefore, the variation in the composition of iron oxide minerals across different regions suggests that the potential for H_2_ production depends on the types and locations of occurrence of BIFs. To obtain more quantified data on the potential of H_2_ release, we attempt to yield quantitative fraction of iron species in the banded iron formation samples using Mössbauer spectroscopy.

### Quantification of iron species in the BIFs: Mössbauer spectroscopy

The Mössbauer spectra for BIF samples can be deconvoluted into multiple sextets and doublets, each characterized by specific hyperfine parameters [i.e., isomer shift (δ), quadrupole splitting (Δ), and hyperfine field (kOe)] that depend on their crystallographic structures [34, 82]. For the minerals with magnetic ordering, such as ferromagnetic magnetite and antiferromagnetic hematite, the sextet in Mössbauer spectra arises due to the magnetic hyperfine fields around iron nuclei. The iron sites in oxide minerals (i.e., tetrahedral and octahedral sites in magnetite, Fe^3+^ in hematite) can be effectively distinguished through hyperfine parameters: for example, the isomer shifts for the tetrahedral (occupied by Fe^3+^, Td^M^) and octahedral [occupied by Fe^2+^ and Fe^3+^ with equal proportion (i.e., Fe^2.5+^), Oh^M^] sites in magnetite are ~0.26 mm/s and ~0.67 mm/s, respectively, and those for octahedral sites in hematite are ~0.37 mm/s [34, 82]. The Td^M^, Oh^M^, and Oh^H^ sites have hyperfine fields of ~490, ~460, and 520 kOe, respectively [34, 82].

**Fig 6:**
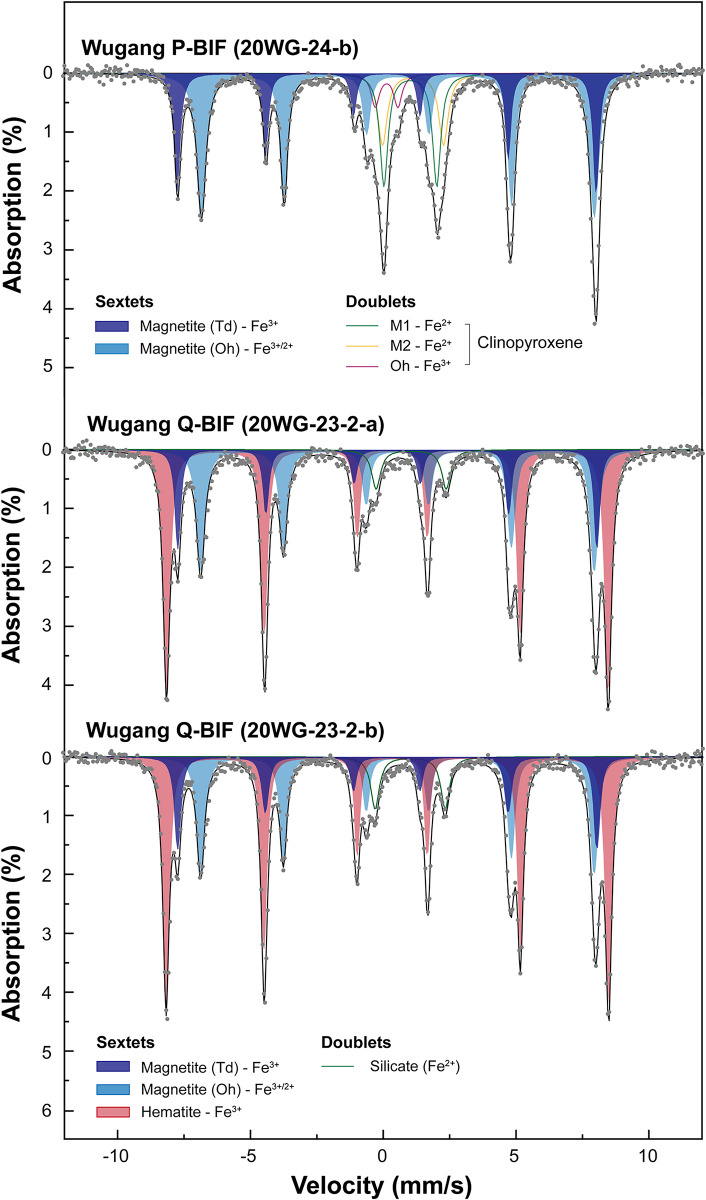
Mössbauer spectra (grey dots) and fitting results (black line) of Wugang BIFs at 298 K. The spectra with three sextets (corresponding to magnetite and hematite) and doublets (corresponding to silicate minerals), as labeled.

**Fig 7:**
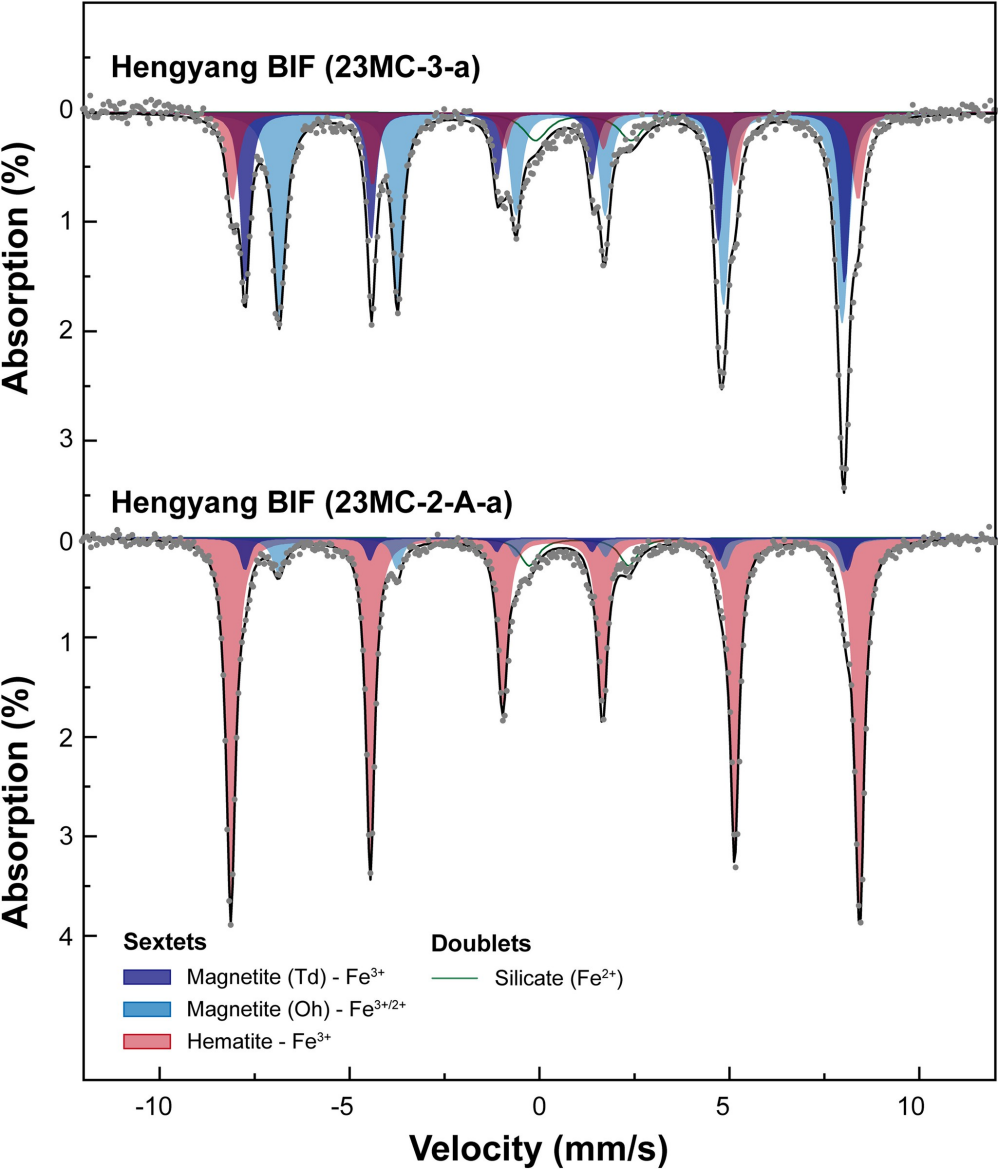
Mössbauer spectra (grey dots) and fitting results (black line) of Hengyang BIFs at 298 K. The spectra with three sextets (corresponding to magnetite and hematite) and doublets (corresponding to silicate minerals), as labeled.

Figs 6 and 7 show the Mössbauer spectra and fitting results for Wugang and Hengyang BIF samples, respectively. The hyperfine parameters for each iron species in varying minerals obtained from the fitting of the experimental data are shown in Table 2. For the Wugang P-BIF (20WG-24-b), the spectrum was deconvoluted with two sextets and three doublets. The former corresponds to the magnetite: the outer sextet with δ = 0.26 mm/s, Δ = 0.00 mm/s, and H_hf_ = 489.8 kOe and the inner sextet with δ = 0.66 mm/s, Δ = 0.00 mm/s, and H_hf_ = 459.5 kOe are assigned to Fe^3+^ tetrahedral site and Fe^2.5+^ octahedral site (which is mixed valence Fe site with both Fe^2+^ and Fe^3+^), respectively. Note that the ratio of area between Td^M^ and Oh^M^ for all samples is ~0.67 (i.e., Td^M^:Oh^M^ = 2:1), which is consistent with the general stoichiometry of magnetite, showing the robustness of the current fitting procedure. The latter is typical characteristics of silicate minerals, consisting of two Fe^2+^ doublets (δ = 1.23 mm/s, Δ = 2.30 mm/s and δ = 1.15 mm/s, Δ = 2.00 mm/s, and one Fe^3+^ doublet (δ = 0.24 mm/s, Δ = 0.85 mm/s)). The doublets can be assigned to clinopyroxene which have three distinct Fe sites (i.e., M1, M2, and Oh^P^ sites) [83]. We note that hematite is not observed in the Mössbauer spectra for P-BIF, which is consistent with the current XRD results. Based on the values of relative area for Oh^M^ with respect to total area of Mössbauer spectra (~52.2%), the calculated Fe^2+^ in magnetite with respect to total iron atoms is about 26.1% in the P-BIF sample. The Fe^2+^ fraction of magnetite relative to total number of iron atoms (Fe^2+^_M_, %) can be calculated as a half of the area of Oh^M^ site, because the Oh^M^ sites occupied by Fe^2+^ and Fe^3+^ with approximately equal proportions [27, 28]. The Mössbauer spectra for Wugang Q-BIF samples (20WG-23-2-a and 20WG-23-2-b) show three sextets corresponding to Td^M^, Oh^M^, and Oh^H^ and one doublet associated with Fe^2+^-bearing silicate mineral. A doublet was fitted with δ = 1.16 mm/s and Δ = 2.63 mm/s for 20WG-23-2-a, and δ = 1.13 mm/s, Δ = 2.63 mm/s for 20WG-23-2-b. Based on the hyperfine parameters, the doublet represents talc observed in XRD patterns [84]. Contrary to P-BIF, the presence of hematite is noticeable in the Mössbauer spectra for Q-BIF samples, consistent with XRD results. The fractions of Fe^2+^ in magnetite for Q-BIFs are ~15–16%, which are considerably lower than those for P-BIF.

**Table 2:** Hyperfine parameters and relative fractions of each iron species obtained from Mössbauer spectroscopy.

Sample	δ (mm/s)^†^	Δ (mm/s) ^‡^	H_hf_ (kOe)^¶^	Relative fraction (%)^§^	Mineral phase	Iron species
*Wugang (WG)*						
20WG-24-b	0.26 (2)	0.00 (2)	489.8 (8)	29.2	Magnetite	Td^M^ (Fe^3+^)
	0.66 (2)	0.00 (2)	459.5 (8)	52.2		Oh^M^ (Fe^2.5+^)
	1.23 (3)	2.30 (3)		6.7	Clinopyroxene	M1 (Fe^2+^)
	1.15 (3)	2.00 (3)		9.1		M2 (Fe^2+^)
	0.24 (2)	0.85 (3)		2.7		Oh^Py^ (Fe^3+^)
20WG-23-2-a	0.36 (2)	-0.09 (2)	517.2 (7)	49.5	Hematite	Oh^H^ (Fe^3+^)
	0.26 (2)	0.00 (2)	489.7 (7)	17.3	Magnetite	Td^M^ (Fe^3+^)
	0.64 (2)	0.00 (2)	459.8 (7)	29.7		Oh^M^ (Fe^2.5+^)
	1.16 (3)	2.63 (3)		3.5	Silicate	Fe^2+^
20WG-23-2-b	0.36 (2)	-0.09 (2)	519.3 (7)	48.7	Hematite	Oh^H^ (Fe^3+^)
	0.26 (2)	0.01 (2)	489.9 (7)	17.0	Magnetite	Td^M^ (Fe^3+^)
	0.64 (2)	0.00 (2)	459.8 (7)	31.7		Oh^M^ (Fe^2.5+^)
	1.13 (3)	2.63 (3)		2.6	Silicate	Fe^2+^
*Hengyang (MC)*						
23MC-3-a	0.37 (2)	-0.10 (2)	511.4 (9)	18.1	Hematite	Oh^H^ (Fe^3+^)
	0.26 (2)	0.00 (2)	489.9 (9)	27.8	Magnetite	Td^M^ (Fe^3+^)
	0.67 (2)	0.00 (2)	459.9 (9)	50.9		Oh^M^ (Fe^2.5+^)
	1.26 (3)	2.51 (3)		3.2	Silicate	Fe^2+^
23MC-2-A-a	0.36 (2)	-0.09 (2)	514.9 (8)	81.9	Hematite	Oh^H^ (Fe^3+^)
	0.26 (2)	0.02 (2)	492.3 (8)	5.9	Magnetite	Td^M^ (Fe^3+^)
	0.67 (2)	0.00 (2)	461.8 (8)	9.5		Oh^M^ (Fe^2.5+^)
	1.15 (3)	2.63 (3)		2.7	Silicate	Fe^2+^

^†^δ: isomer shifts (mm/s). Note that isomer shifts are reported relative to α-Fe foil at 298 K

^‡^Δ: quadrupole splitting (mm/s)

^¶^H_hf_: hyperfine field (kOe)

^§^The fractions of each iron species were estimated from the relative area of sextets or doublets representing each iron species in Mössbauer spectra. The uncertainty for relative fraction is ~ ±1.0–2.0%.

Note: Number in parentheses denotes uncertainties of Mössbauer parameters such as δ, Δ, and H_hf_ [e.g., 0.26 (2) = 0.26 ± 0.02]

The Mössbauer spectra for BIF samples from the Hengyang region are also deconvoluted into two sextets for magnetite, one sextet for hematite, and one doublet for Fe^2+^-bearing silicate mineral, while the relative area of each component varies among samples. Detailed fitting parameters are presented in Table 2. For 23MC-3-a, the relative fraction of the sextet for octahedral site of magnetite (Fe^2.5+^) is approximately 50.9%, indicating that Fe^2+^ in magnetite (Fe^2+^_M_) is about 25.5%. Notably, the predominance of hematite (~81.9%) are observed in the 23MC-2-A-a, indicating the lack of magnetite in this sample.

### Magnetite in BIFs: The potential source for H_2_

Recent pioneering study has explored the potential process for natural H_2_ generation via weathering and alteration of natural BIFs with aqueous fluids, based on the systematic analysis of their petrological, mineralogical, and geochemical characteristics [24]. This study suggests that the oxidation of Fe^2+^ in ferrous minerals including magnetite, can release natural H_2_, occurring near the surface under low-temperature conditions. Furthermore, the several water-rock interaction experiments have reported that the magnetite can produce considerable amounts of H_2_ in relatively lower temperature conditions (< 100°C) than hydrothermal processes such as serpentinization [25, 30]. These studies indicate that magnetite can be a main source of hydrogen both in naturally released and stimulated production.

Based on the generic redox Eq (1) that describes iron oxidation and H_2_ generation [25], the potential for H₂ production from Fe^2^⁺ in magnetite can be estimated. The mechanism involves a redox reaction occurring in anoxic environments, where water oxidizes Fe^2+^ to Fe^3+^ and is itself reduced to form H₂. Furthermore, the oxidation of magnetite within BIFs to hematite, induced by water-rock interaction, plays the crucial role in promoting H_2_ production in this process.


$$2Fe^{2+}O_{magnetite}+H_{2}O\Leftrightarrow F{e^{3+}}_{2}O_{3hematite}+H_{2}$$ (1)

To estimate the potential of H_2_ release from magnetite in the BIFs, quantification of the fractions of individual iron species using Mössbauer spectroscopy is necessary. Table 3 shows the total Fe contents in bulk sample (Fe^T^, wt%), the relative fraction of Fe^2+^ in magnetite (Fe^2+^_Mag_, %) and that of Fe atoms in magnetite with respect to total iron atoms (Fe_Mag_, which is the sum of Fe^2+^ and Fe^3+^, %), and the proportion of magnetite in the bulk sample (*X*_Mag_, wt%). Here, *n* typically represents the number of moles, while *X* is used in a chemical context to denote either a percentage or a weight fraction. As mentioned in above, the Fe^2+^_Mag_ can be obtained by the half of Oh^M^ area, and Fe_Mag_ can be calculated by the sum of areas for Oh^M^ and Td^M^. Note that *X*_Mag_ (wt%) can be calculated by multiplying Fe^T^ (wt%) by Fe_Mag_ (%) and then dividing by 0.72, where 0.72 represents the ratio of the mass of iron atoms to the total mass of Fe₃O₄, accounting for the three Fe atoms present in each formula unit of magnetite. The maximum H_2_ potentials (mmol/kg) which can be produced from Fe^2+^ in magnetite were obtained by following the previously reported calculation steps [25]. Based on Eq (1), one mole of H₂ can be produced for every two moles of Fe^2^⁺O in magnetite, indicating the necessity to determine the number of moles of Fe^2^⁺ in magnetite within 1 kg of bulk BIF samples (*n*FeO_Mag_). The weight percent of Fe^2^⁺ in magnetite (*X*Fe^2+^_Mag_) relative to the bulk sample is calculated by multiplying the total Fe content (Fe^T^, wt%) by the percentage of Fe^2^⁺ in magnetite (Fe^2+^_Mag_, %). The weight percent of Fe^2+^O in magnetite (*X*Fe^2+^O_Mag_) is then obtained by multiplying *X*Fe^2+^_Mag_ by 0.78, which represents the molar mass ratio of Fe to FeO. Using the molar mass of FeO (71.844 g/mol), *X*Fe^2+^O_Mag_ is further converted to moles of *n*FeO_Mag_ per unit mass of rock. Finally, the number of moles of H_2_ can be determined by dividing *n*FeO_Mag_ by 2 and then converted to millimoles in kilogram to obtain the final result [25].

**Table 3:** Total Fe contents (Fe^T^, wt%), the fraction of Fe^2+^ in magnetite (Fe2+Mag, %), the fraction of Fe atoms in magnetite relative to total Fe atoms in the bulk sample (Fe_Mag_, %), and the proportion of magnetite in the bulk sample (*X*_Mag_, %). The iron redox state of the bulk sample (Fe^3+^/Fe_tot_) and estimated H_2_ (mmol/kg) potential are also presented.

Sample	Fe^T^ (wt%)^a^	Fe^2+^_Mag_ (%)^b^	Fe^3+^_Mag_ (%)^c^	Fe_Mag_ (%)^d^	*X*_Mag_^e^ (wt%)	Fe^3+^/Fe_tot_ (bulk)	H_2_ potential (mmol/kg)
*Wugang (WG)*							
20WG-24-b	27.0	26.1	55.3	81.4	30.5	0.68	628.9
20WG-23-2-a	34.3	14.9	32.2	47.0	22.4	0.85	455.0
20WG-23-2-b	31.2	15.9	32.9	48.7	21.1	0.84	440.6
*Hengyang (MC)*							
23MC-3-a	21.4	25.5	53.3	78.7	23.4	0.74	486.6
23MC-2-A-a	28.3	4.7	10.7	15.4	6.0	0.95	119.4

^a^ Total Fe (Fe^T^) values were calculated from Fe_2_O_3_^T^ (wt%) measured by ICP-OES, with an error range of approximately ±0.1 wt%.

^b^ The fraction of Fe^2+^ species in magnetite with respect to total Fe atoms in bulk sample was a half of the Oh^M^.

^c^ The fraction of Fe^3+^ species in magnetite with respect to total Fe atoms in bulk sample was combined with half of the Oh^M^ and the Td^M^.

^d^ The fraction of Fe in magnetite with respect to total Fe atoms obtained from the sum of Td^M^ and Oh^M^ (see Table 2).

^e^ The contents of magnetite in bulk sample were calculated utilizing total Fe_2_O_3_ contents (wt%, see Table 1) and Fe_Mag_ (%) fraction relative to total iron atoms.

The P-BIF sample from Wugang (20WG-24-b), which composed of predominantly magnetite, showed low Fe^3^⁺/Fe_tot_ ratio (~0.68) and high content of Fe^2^⁺ in magnetite (~26%). The estimated potential of H_2_ production of magnetite-rich BIFs in China Craton is ~630 mmol H₂/kg rock. Meanwhile, the hematite-rich BIF sample from Hengyang (i.e., 23MC-2-A-a) have significantly lower Fe^2+^ fraction in magnetite (~5%), resulting in low H_2_ potential (~120 mmol H₂/kg rock). Note that the unaltered BIFs from the Hamersley Province of Western Australia are thermodynamically estimated to produce 200 mmol H_2_/kg [25]. Compared to the previous study, the secondary minerals such as goethite or maghemite were not observed, indicating that the BIFs in China Craton have significant potential to produce H_2_ because these BIFs are rather fresh. The current results showed that the magnetite-rich BIF can be a probable candidate for H_2_ production and H_2_ generation potential varies with mineralogical compositions depending on the types and locations of occurrence of BIFs. The results of this study highlight that it is essential to prioritize the exploration of deposits with favorable geochemical and mineralogical conditions, particularly those with a dominance of magnetite. Experimental techniques, including Mössbauer spectroscopy, are effective for accurately quantifying iron species and providing details of mineral compositions from diverse geological settings. Additionally, laboratory experiments simulating subsurface conditions with natural BIF samples could further elucidate the reaction mechanisms and kinetics associated with hydrogen release, contributing to optimization of conditions for maximizing H_2_ production. Establishing a mineralogical database focused on the relative fractions of iron species across various BIFs would also be valuable for guiding future exploration and resource development.

## Conclusions

In this study, we investigated the mineralogical and geochemical characteristics of natural iron ore samples from the Wugang and Hengyang BIF in China. Specifically, we identified the mineral phases in the BIF samples and quantified the individual iron species in varying iron-bearing phases including magnetite and hematite, using XRD and Mössbauer spectroscopy. The current results allow us to evaluate the potential of H_2_ production from Fe^2+^ species in the magnetite of BIFs. The details of mineral compositions in BIF vary with the types and locations of occurrence: particularly, the pyroxene-bearing BIF samples collected in Wugang showed the predominance of magnetite, while the hematite is dominant in the BIF samples from Hengyang region. The maximum potential of H_2_ production of the sample with high content of Fe^2+^ in magnetite concerning total iron atoms (~26%) was calculated to be ~ 630 mmol H₂/kg rock, indicating that magnetite-rich BIF could be promising candidates for H_2_ production. The current study highlights that the Mössbauer spectroscopy can be effectively utilized to characterize the geological source rocks for the future exploration of H_2_ production sites. We believe that our findings can contribute to establish the mineralogical database of BIFs in the China Craton, providing a basis for assessing their potential for H_2_ production.

## References

[1] Nikolaidis P, Poullikkas A. A comparative overview of hydrogen production processes. Renew Sustain Energy Rev. 2017;67:597–611.

[2] Smith N, Shepherd T, Styles M, Williams G, editors. Hydrogen exploration: a review of global hydrogen accumulations and implications for prospective areas in NW Europe. Geological Society, London, Petroleum Geology Conference Series; 2005: The Geological Society of London.

[3] Zgonnik V. The occurrence and geoscience of natural hydrogen: A comprehensive review. Earth-Sci Rev. 2020;203:103140.

[4] Prinzhofer A, Cissé CST, Diallo AB. Discovery of a large accumulation of natural hydrogen in Bourakebougou (Mali). Int J Hydrogen Energy. 2018;43(42):19315–26.

[5] Boulart C, Chavagnac V, Monnin C, Delacour A, Ceuleneer G, Hoareau G. Differences in gas venting from ultramafic-hosted warm springs: the example of Oman and Voltri ophiolites. Ofioliti. 2013;38(2):143–56.

[6] Templeton AS, Ellison ET, Kelemen PB, Leong J, Boyd ES, Colman DR, et al. Low-temperature hydrogen production and consumption in partially-hydrated peridotites in Oman: implications for stimulated geological hydrogen production. Front Geochem. 2024;2:1366268.

[7] Vacquand C, Deville E, Beaumont V, Guyot F, Sissmann O, Pillot D, et al. Reduced gas seepages in ophiolitic complexes: Evidences for multiple origins of the H2-CH4-N2 gas mixtures. Geochim Cosmochim Acta. 2018;223:437–61.

[8] Guélard J, Beaumont V, Rouchon V, Guyot F, Pillot D, Jézéquel D, et al. Natural H_2_ in K ansas: Deep or shallow origin? Geochem Geophys Geosyst. 2017;18(5):1841–65.

[9] Osselin F, Soulaine C, Fauguerolles C, Gaucher E, Scaillet B, Pichavant M. Orange hydrogen is the new green. Nature Geosci. 2022;15(10):765–9.

[10] Crouzet C, Brunet F, Recham N, Findling N, Lanson M, Guyot F, et al. Hydrogen production by hydrothermal oxidation of FeO under acidic conditions. Int J Hydrogen Energy. 2017;42(2):795–806.

[11] Klein C. Some Precambrian banded iron-formations (BIFs) from around the world: Their age, geologic setting, mineralogy, metamorphism, geochemistry, and origins. Am Mineral. 2005;90(10):1473–99.

[12] Albers E, Bach W, Pérez-Gussinyé M, McCammon C, Frederichs T. Serpentinization-driven H2 production from continental break-up to mid-ocean ridge spreading: unexpected high rates at the West Iberia margin. Front Earth Sci. 2021;9:673063.

[13] McCollom TM, Bach W. Thermodynamic constraints on hydrogen generation during serpentinization of ultramafic rocks. Geochim Cosmochim Acta. 2009;73(3):856–75.

[14] Baciu C, Etiope G. A direct observation of a hydrogen-rich pressurized reservoir within an ophiolite (Tișovița, Romania). Int J Hydrogen Energy. 2024;73:402–6.

[15] Etiope G. Natural hydrogen extracted from ophiolitic rocks: A first dataset. Int J Hydrogen Energy. 2024;78:368–72.

[16] Truche L, Donzé F-V, Goskolli E, Muceku B, Loisy C, Monnin C, et al. A deep reservoir for hydrogen drives intense degassing in the Bulqizë ophiolite. Science. 2024;383(6683):618–21. 38330123 10.1126/science.adk9099

[17] Combaudon V, Moretti I, Kleine BI, Stefánsson A. Hydrogen emissions from hydrothermal fields in Iceland and comparison with the Mid-Atlantic Ridge. Int J Hydrogen Energy. 2022;47(18):10217–27.

[18] Jt Welhan, Craig H. Methane and hydrogen in East Pacific Rise hydrothermal fluids. Geophys Res Lett. 1979;6(11):829–31.

[19] Worman SL, Pratson LF, Karson JA, Schlesinger WH. Abiotic hydrogen (H2) sources and sinks near the Mid-Ocean Ridge (MOR) with implications for the subseafloor biosphere. Proceedings of the National Academy of Sciences. 2020;117(24):13283–93. 10.1073/pnas.2002619117 PMC7306814 32482880

[20] Arnórsson S. Gas pressures in geothermal systems. Chem Geol. 1985;49(1–3):319–28.

[21] Charlou JL, Donval JP, Konn C, Ondréas H, Fouquet Y, Jean-Baptiste P, et al. High production and fluxes of H2 and CH4 and evidence of abiotic hydrocarbon synthesis by serpentinization in ultramafic-hosted hydrothermal systems on the Mid-Atlantic Ridge. Geophys Monogr. 2010;188:265–96.

[22] Quéméneur M, Mei N, Monnin C, Postec A, Guasco S, Jeanpert J, et al. Microbial taxa related to natural hydrogen and methane emissions in serpentinite-hosted hyperalkaline springs of New Caledonia. Front Microbiol. 2023;14:1196516. doi: 10.3389/fmicb.2023.1196516 37485525 PMC10359428

[23] Blay-Roger R, Bach W, Bobadilla LF, Reina TR, Odriozola JA, Amils R, et al. Natural hydrogen in the energy transition: Fundamentals, promise, and enigmas. Renew Sustain Energy Rev. 2024;189:113888.

[24] Geymond U, Briolet T, Combaudon V, Sissmann O, Martinez I, Duttine M, et al. Reassessing the role of magnetite during natural hydrogen generation. Front Earth Sci. 2023;11:1169356.

[25] Geymond U, Ramanaidou E, Lévy D, Ouaya A, Moretti I. Can weathering of banded iron formations generate natural hydrogen? Evidence from Australia, Brazil and South Africa. Miner. 2022;12(2):163.

[26] Bekker A, Slack JF, Planavsky N, Krapez B, Hofmann A, Konhauser KO, et al. Iron formation: the sedimentary product of a complex interplay among mantle, tectonic, oceanic, and biospheric processes. Econ Geol. 2010;105(3):467–508.

[27] Lindsley DH. The crystal chemistry and structure of oxide minerals as exemplified by the Fe-Ti oxides. Oxide minerals. 1976;3:L1–L60.

[28] Waychunas GA. Crystal chemistry of oxides and oxyhydroxides. Rev Mineral Geochem. 1991;25(1):11–68.

[29] Irfan M, Zhou L, Bai Y, Yuan S, Liang T-T, Liu Y-F, et al. Insights into the hydrogen generation from water-iron rock reactions at low temperature and the key limiting factors in the process. Int J Hydrogen Energy. 2019;44(33):18007–18.

[30] Mayhew LE, Ellison E, McCollom T, Trainor T, Templeton A. Hydrogen generation from low-temperature water–rock reactions. Nat Geosci. 2013;6(6):478–84.

[31] Abdel-Hakeem M, El-Habaak G. Textural complications of banded iron formation and the potential production of nano-magnetite: a case study from the Central Eastern Desert of Egypt. Sci Rep. 2023;13(1):15158. doi: 10.1038/s41598-023-42058-5 37704678 PMC10499896

[32] Posth NR, Köhler I, Swanner ED, Schröder C, Wellmann E, Binder B, et al. Simulating Precambrian banded iron formation diagenesis. Chem. Geol. 2013;362:66–73.

[33] Sun S, Li Y-L. Geneses and evolutions of iron-bearing minerals in banded iron formations of> 3760 to ca. 2200 million-year-old: Constraints from electron microscopic, X-ray diffraction and Mössbauer spectroscopic investigations. Precambrian Res. 2017;289:1–17.

[34] Dyar MD, Agresti DG, Schaefer MW, Grant CA, Sklute EC. Mössbauer spectroscopy of earth and planetary materials. Annu Rev Earth Planet Sci. 2006;34(1):83–125.

[35] McCammon CA. Insights into phase transformations from Mössbauer spectroscopy. Rev Mineral Geochem. 2000;39(1):241–64.

[36] Gunnlaugsson HP, Rasmussen H, Madsen M, Nørnberg P. New analysis of the Mössbauer spectra of olivine basalt rocks from Gusev crater on Mars. Planet Space Sci. 2009;57(5–6):640–5.

[37] Hao X-L, Li Y-L. ^57^Fe Mössbauer spectroscopy of mineral assemblages in mantle spinel lherzolites from Cenozoic alkali basalt, eastern China: Petrological applications. Lithos. 2013;156:112–9.

[38] Harding SC, Nash BP, Petersen EU, Ekdale A, Bradbury CD, Dyar MD. Mineralogy and geochemistry of the Main Glauconite Bed in the middle Eocene of Texas: paleoenvironmental implications for the verdine facies. PloS one. 2014;9(2):e87656. doi: 10.1371/journal.pone.0087656 24503875 PMC3913656

[39] Uhm YR, Sun G-M, Jin M-E, Jwa Y-J, Seo JY, Choi H, et al. Provenance Studies for Prehistoric Obsidian by Using Mössbauer Spectroscopy. J Korean Phys Soc. 2020;77:253–7.

[40] Dunlap R, McGraw J. A Mössbauer effect study of Fe environments in impact glasses. J Non-Cryst Solids. 2007;353(22–23):2201–5.

[41] Mysen BO. The structural behavior of ferric and ferrous iron in aluminosilicate glass near meta-aluminosilicate joins. Geochim Cosmochim Acta. 2006;70(9):2337–53.

[42] Li L-M, Sun M, Wang Y, Xing G, Zhao G, Lin S, et al. U–Pb and Hf isotopic study of zircons from migmatised amphibolites in the Cathaysia Block: implications for the early Paleozoic peak tectonothermal event in Southeastern China. Gondwana Res. 2011;19(1):191–201.

[43] Nayak P, Das D, Singh P, Chakravortty V. ^57^Fe Mössbauer spectroscopy of banded iron formations from eastern India. J Radioanal Nucl Chem. 2004;260(1):19–26.

[44] Orberger B, Wagner C, Tudryn A, Wirth R, Morgan R, Fabris JD, et al. Micro-to nano-scale characterization of martite from a banded iron formation in India and a lateritic soil in Brazil. Phys Chem Miner. 2014;41:651–67.

[45] Salama W, El Aref M, Gaupp R. Spectroscopic characterization of iron ores formed in different geological environments using FTIR, XPS, Mössbauer spectroscopy and thermoanalyses. Spectrochim Acta A Mol Biomol Spectrosc. 2015;136:1816–26. 25467675 10.1016/j.saa.2014.10.090

[46] Huang J, Jones A, Waite TD, Chen Y, Huang X, Rosso KM, et al. Fe (II) redox chemistry in the environment. Chem Rev. 2021;121(13):8161–233. doi: 10.1021/acs.chemrev.0c01286 34143612

[47] Li H, He J, Liang J, Yang F, Zhai M, Zhang L, et al. Geochemical characteristics of Wuyang siliceous rocks in the southern margin of North China Craton and its constraint on the formation environment of BIF of Tieshanmiao Formation. Acta Geol Sin. 2019;93(6):1738–54.

[48] Liu L, Zhang H, Yang X, Li Y. Age, origin and significance of the Wugang BIF in the Taihua complex, Southern North China Craton. Ore Geol Rev. 2018;95:880–98.

[49] Zhou H, Zhou W, Wei Y, Fru EC, Huang B, Fu D, et al. Mesoarchean banded iron-formation from the northern Yangtze Craton, South China and its geological and paleoenvironmental implications. Precambrian Res. 2022;383:106905.

[50] Zhang S, Chang L, Zhao H, Ding J, Xian H, Li H, et al. The Precambrian drift history and paleogeography of the Chinese cratons. Ancient supercontinents and the paleogeography of Earth: Elsevier; 2021. 333–76.

[51] Liu D, Nutman A, Compston W, Wu J, Shen QH. Remnants of≥ 3800 Ma crust in the Chinese part of the Sino-Korean craton. Geology. 1992;20(4):339–42.

[52] Song B, Nutman AP, Liu D, Wu J. 3800 to 2500 Ma crustal evolution in the Anshan area of Liaoning Province, northeastern China. Precambrian Res. 1996;78(1–3):79–94.

[53] Zheng J, Griffin W, O’Reilly SY, Lu F, Wang C, Zhang M, et al. 3.6 Ga lower crust in central China: new evidence on the assembly of the North China Craton. Geology. 2004;32(3):229–32.

[54] Duan H, Wang C, Shi K, Wang C, Chen Q, Zhu J, et al. Insights into characterization and genesis of the Tieshanmiao banded iron formation deposit, China: Evidence from zircon U–Pb dating and geochemistry. Ore Geol Rev. 2021;138:104329.

[55] Jishun R. On the geotectonics of southern China. Acta Geol Sin. 1991;4(2):111–30.

[56] Wong J, Sun M, Xing G, Li X-h, Zhao G, Wong K, et al. Zircon U–Pb and Hf isotopic study of Mesozoic felsic rocks from eastern Zhejiang, South China: geochemical contrast between the Yangtze and Cathaysia blocks. Gondwana Res. 2011;19(1):244–59.

[57] Ames L, Gaozhi Z, Baocheng X. Geochronology and isotopic character of ultrahigh‐pressure metamorphism with implications for collision of the Sino‐Korean and Yangtze cratons, central China. Tectonics. 1996;15(2):472–89.

[58] Qiu YM, Gao S, McNaughton NJ, Groves DI, Ling W. First evidence of> 3.2 Ga continental crust in the Yangtze craton of south China and its implications for Archean crustal evolution and Phanerozoic tectonics. Geology. 2000;28(1):11–4.

[59] Chen J, Jahn B-m. Crustal evolution of southeastern China: Nd and Sr isotopic evidence. Tectonophysics. 1998;284(1–2):101–33.

[60] Li X-h, Li Z-X, Zhou H, Liu Y, Kinny PD. U–Pb zircon geochronology, geochemistry and Nd isotopic study of Neoproterozoic bimodal volcanic rocks in the Kangdian Rift of South China: implications for the initial rifting of Rodinia. Precambrian Res. 2002;113(1–2):135–54.

[61] Rino S, Kon Y, Sato W, Maruyama S, Santosh M, Zhao D. The Grenvillian and Pan-African orogens: world’s largest orogenies through geologic time, and their implications on the origin of superplume. Gondwana Res. 2008;14(1–2):51–72.

[62] Santosh M, Maruyama S, Yamamoto S. The making and breaking of supercontinents: some speculations based on superplumes, super downwelling and the role of tectosphere. Gondwana Res. 2009;15(3–4):324–41.

[63] Zhao G, Wilde SA, Cawood PA, Sun M. Archean blocks and their boundaries in the North China Craton: lithological, geochemical, structural and P–T path constraints and tectonic evolution. Precambrian Res. 2001;107(1–2):45–73.

[64] Lan C, Zhang L, Zhao T, Wang C, Li H, Zhou Y. Mineral and geochemical characteristics of the Tieshanmiao-type BIF-iron deposit in Wuyang region of Henan Province and its implications for ore-forming processes. Acta Geol Sin. 2013;29(7):2567–82.

[65] Santosh M. Assembling North China Craton within the Columbia supercontinent: the role of double-sided subduction. Precambrian Res. 2010;178(1–4):149–67.

[66] Li J, Zhang Y, Zhao G, Johnston ST, Dong S, Koppers A, et al. New insights into Phanerozoic tectonics of South China: Early Paleozoic sinistral and Triassic dextral transpression in the east Wuyishan and Chencai domains, NE Cathaysia. Tectonics. 2017;36(5):819–53.

[67] Yao T, Li H-M, Li W-J, Li L-X, Zhao C. Origin of the disseminated magnetite pyroxenite in the Tieshanmiao-type iron deposits in the Wuyang region of Henan Province, China. J Asian Earth Sci. 2015;113:1235–52.

[68] Yan Y, Hu X-q, Lin G, Santosh M, Chan L-S. Sedimentary provenance of the Hengyang and Mayang basins, SE China, and implications for the Mesozoic topographic change in South China Craton: Evidence from detrital zircon geochronology. J Asian Earth Sci. 2011;41(6):494–503.

[69] Yin C-Y LP-J, Tang F, Gao L-Z, Yang Z-Q, Song B. New U-Pb zircon age constrains on the age of the interglacial Fulu Formation in Zhaoxing, Liping, Guizhou, South China. 33_rd_ Intenational Geological Congress. 2008.

[70] Moon I, Lee I, Yang X. Geochemical Studies of BIF in Wugang, North China Craton: Implication for the Genesis. Econ Environ Geol. 2019;52(3):213–21.

[71] Kato Y, Kawakami T, Kano T, Kunugiza K, Swamy N. Rare-earth element geochemistry of banded iron formations and associated amphibolite from the Sargur belts, south India. Journal of Southeast Asian Earth Sciences. 1996;14(3–4):161–4.

[72] Bolhar R, Kamber BS, Moorbath S, Fedo CM, Whitehouse MJ. Characterisation of early Archaean chemical sediments by trace element signatures. Earth Planet Sci Lett. 2004;222(1):43–60.

[73] He H, Zhong Y, Liang X, Tan W, Zhu J, Yan Wang C. Natural Magnetite: an efficient catalyst for the degradation of organic contaminant. Sci Rep. 2015;5(1):10139. doi: 10.1038/srep10139 25958854 PMC4426601

[74] Jean M, Nachbaur V, Le Breton J-M. Synthesis and characterization of magnetite powders obtained by the solvothermal method: influence of the Fe^3+^ concentration. J Alloys Compd. 2012;513:425–9.

[75] Pap MSAS. Crystal-chemical characterization of clinopyroxenes based on eight new structure refinements. 1969.

[76] Levien L, Prewitt CT, Weidner DJ. Structure and elastic properties of quartz at pressure. Am Mineral. 1980;65(9–10):920–30.

[77] Liu X, Qiu G, Yan A, Wang Z, Li X. Hydrothermal synthesis and characterization of α-FeOOH and α-Fe_2_O_3_ uniform nanocrystallines. J Alloys Compd. 2007;433(1–2):216–20.

[78] Wang S-B, Min Y-L, Yu S-H. Synthesis and magnetic properties of uniform hematite nanocubes. J Phys Chem C. 2007;111(9):3551–4.

[79] Bailey S. Refinement of an intermediate microcline structure. Am Min. 1969;54(11–12):1540–5.

[80] Fitz Gerald JD, Parise JB, Mackinnon ID. Average structure of an An_48_ plagioclase from the Hogarth Ranges. Am Mineral. 1986;71(11–12):1399–408.

[81] Drits VA, Guggenheim S, Zviagina BB, Kogure T. Structures of the 2: 1 layers of pyrophyllite and talc. CCM 2012;60(6):574–87.

[82] Murad E. Magnetic properties of microcrystalline iron (III) oxides and related materials as reflected in their Mössbauer spectra. Phys Chem Miner. 1996;23(4):248–62.

[83] De Grave E, Eeckhout SG. ^57^Fe Mossbauer-effect studies of Ca-rich, Fe-bearing clinopyroxenes: Part III. Diopside. Am Min. 2003;88(7):1145–52.

[84] Corona JC, Jenkins DM, Dyar MD. The experimental incorporation of Fe into talc: a study using X-ray diffraction, Fourier transform infrared spectroscopy, and Mössbauer spectroscopy. Contrib Mineral Petrol. 2015;170:1–15.

